# Salinity pretreatment synergies heat shock toxicity in cyanobacterium *Anabaena* PCC7120

**DOI:** 10.3389/fmicb.2023.1061927

**Published:** 2023-02-16

**Authors:** Rupanshee Srivastava, Tripti Kanda, Sadhana Yadav, Nidhi Singh, Shivam Yadav, Rajesh Prajapati, Vigya Kesari, Neelam Atri

**Affiliations:** ^1^Department of Botany, Institute of Sciences, Banaras Hindu University, Varanasi, India; ^2^Department of Botany, Thakur Prasad Singh (T.P.S.) College, Patna, Bihar, India; ^3^Department of Botany, Mahila Mahavidyalaya (M.M.V.), Banaras Hindu University, Varanasi, India

**Keywords:** cyanobacteria, *Anabaena* PCC7120, salinity, heat shock, pretreatments, stress

## Abstract

This study was undertaken to bridge the knowledge gap pertaining to cyanobacteria’s response to pretreatment. The result elucidates the synergistic effect of pretreatment toxicity in cyanobacterium *Anabaena* PCC7120 on morphological and biochemical attributes. Chemical (salt) and physical (heat) stress-pretreated cells exhibited significant and reproducible changes in terms of growth pattern, morphology, pigments, lipid peroxidation, and antioxidant activity. Salinity pretreatment showed more than a five-fold decrease in the phycocyanin content but a six-fold and five-fold increase in carotenoid, lipid peroxidation (MDA content), and antioxidant activity (SOD and CAT) at 1 h and on 3rd day of treatment, respectively, giving the impression of stress-induced free radicals that are scavenged by antioxidants when compared to heat shock pretreatment. Furthermore, quantitative analysis of transcript (qRT-PCR) for FeSOD and MnSOD displayed a 3.6- and 1.8-fold increase in salt-pretreated (S-H) samples. The upregulation of transcript corresponding to salt pretreatment suggests a toxic role of salinity in synergizing heat shock. However, heat pretreatment suggests a protective role in mitigating salt toxicity. It could be inferred that pretreatment enhances the deleterious effect. However, it further showed that salinity (chemical stress) augments the damaging effect of heat shock (physical stress) more profoundly than physical stress on chemical stress possibly by modulating redox balance *via* activation of antioxidant responses. Our study reveals that upon pretreatment of heat, the negative effect of salt can be mitigated in filamentous cyanobacteria, thus providing a foundation for improved cyanobacterial tolerance to salt stress.

## 1. Introduction

Anthropogenic activities in agricultural fields such as overuse of chemical fertilizers and improper irrigation practices increase the salt and metal contents in the soil. Furthermore, an increase in greenhouse gas emissions leads to the accumulation of heat-trapping gases in the atmosphere and thus warms the climate, which eventually disturbs the soil ecosystem as well as soil microflora, particularly cyanobacteria. Thus, salinity and heat are considered agriculturally relevant abiotic stresses and are of particular interest. Cyanobacteria are photosynthetic prokaryotes, present in extreme environments, and serve as a dominant producer in paddy fields. They tolerate unfavorable environmental conditions and thus have evolved various responses to acclimatize to stresses (Wannigama et al., 2012). *Anabaena*, a filamentous nitrogen-fixing cyanobacterium, is known to fix a significant amount of nitrogen in Indian paddy fields. However, in paddy fields, they are exposed to various abiotic stresses, including heat and salinity. Salt stress is an abiotic element that has a huge impact on cyanobacterial survival and growth. In cells of photosynthetic organisms, salt stress leads to a decrease in cell volume and an increase in osmotic stress (Yang et al., 2020). It also inhibits processes like photosynthesis, protein synthesis, energetics, and lipid metabolism. Changes in ion and water homeostasis due to salt cause damage at the molecular level, arrest growth, and can even cause death (Demiral and Türkan, 2006). The low concentration of Na^+^ aids in the regulation of pH and nitrogen and carbon dioxide fixation in plants and cyanobacteria (Karandashova and Elanskaya, 2005; Han et al., 2012; Fan et al., 2019). Ion homeostasis is disrupted by excessive Na^+^ and Cl^–^ fluxes into the cell, resulting in the build-up of ROS (Scarpeci et al., 2008; Feng et al., 2015; Song et al., 2020). ROS leads to the breakdown of the photosynthetic machinery and membrane lipid peroxidation, which has a negative impact on photosynthesis (Yang et al., 2020). Furthermore, high NaCl concentrations prevent the *de novo* synthesis of proteins, including several photosystem-related proteins like the D1 protein (Allakhverdiev et al., 2002).

However, heat damages the cellular membrane and leads to oxidative damage, thereby producing ROS such as superoxide anions, hydroxyl radicals, and peroxides (Liu and Huang, 2000). Furthermore, it is also known to inhibit growth, photosynthesis, metabolism, and RNA synthesis and induce protein denaturation and aggregation causing post-translational modifications leading to the loss of cell viability (Wen et al., 2005). Photosystem II (PSII) is regarded to be one of the most sensitive locations in photosynthetic machinery, and its activity is considerably reduced by high-temperature stress (Zhao et al., 2008).

However, most of the studies concerning stress are of single stress type (Rai et al., 2013). The pretreatment effect is distinctive and cannot be extrapolated from the responses to each of them when applied alone. It is reasonable to presume that the molecular signaling pathway and the complex network of interrelated genes may synergize and/or antagonize each other’s effects (Rai et al., 2013). Even though the pretreatment phenomenon has not yet been reported to exist in the environment but it can be applied to various biotechnological approaches, as mentioned in the study of Ellison et al. (2019) in where they studied the effect of pretreatment for enhanced lipid extraction. Pretreatment studies of salt, temperature, and copper followed by UV stress in *Anabaena doliolum* (Srivastava et al., 2006), pretreatment of copper followed by UV stress in *Anabaena doliolum* (Bhargava et al., 2008), and heat pretreatment followed by UV stress in *Anabaena doliolum* (Mishra et al., 2009) are one of the very few reports on cyanobacteria pertaining to pretreatment.

A thorough review of the literature has revealed the availability of facts of cyanobacteria subjected to single stresses as well as in combination (Narayan et al., 2011; Pandey et al., 2012; Rai et al., 2013, 2014; Agrawal et al., 2014; Panda et al., 2014; Shrivastava et al., 2015; Singh et al., 2015; Teikari et al., 2015). However, studies pertaining to pretreatments are very limiting. Furthermore, heat treatment has been found to offer cross-tolerance to different abiotic stresses such as salt (Mishra et al., 2009) but studies pertaining to the effects of salt (chemical stress) on heat shock (physical stresses) is limiting. Therefore, this study was conducted to bridge the above-mentioned gap in the knowledge regarding the response of cyanobacteria subjected to pretreatment. In light of this and the physicochemical characteristics of the relevant stresses, we postulated that salinity pretreatment should enhance the heat effect while heat pretreatment should counteract the salinity effect even if applied after 7 days. To test the aforementioned hypotheses, experiments were conducted to investigate the effect of pretreatment with salt and temperature on the morphology, pigment content, lipid peroxidation (MDA content), enzymatic (superoxide dismutase and catalase) modifications, and expression pattern of antioxidative genes in *Anabaena* PCC7120.

## 2. Materials and methods

### 2.1. Organism and growth condition

*Anabaena* PCC7120 was grown axenically in BG-11 medium (Rippka et al., 1979) buffered with 4-(2-hydroxyethyl)-1-piperazine-ethanesulfonic acid (HEPES) buffer (1.2 g L^–1^) at pH 7.5 in Erlenmeyer flask of capacity 250 ml containing 100 ml of culture at 24 ± 2*^o^*C under a day light fluorescent tube emitting 72 μmol m^–2^ s^–1^ photosynthetically active radiation (PAR) light intensity and a photoperiod of 14:10 h. The flasks were plugged with non-absorbent cotton and were shaken 2–3 times daily for proper aeration. All experiments were performed under an exponentially growing culture.

### 2.2. Experimental design and treatments

*Anabaena* PCC7120 culture (OD_750_ = 0.5) never exposed to stress was taken as control strain. The single, combined, and pretreatment of heat and salt treatment were given to the strain at LC_50_ (lethal concentration). LC_50_ was determined through the colony count method (Rai and Raizada, 1985).

#### 2.2.1. For LC_50_NaCl determination

Exponentially growing *Anabaena* containing 10^4^ cells mL^–1^ was aseptically spread on agar plates supplemented with various concentrations of NaCl (50–250 mM).

#### 2.2.2. For LC_50_ heat shock determination

Exponentially growing *Anabaena* containing 10^4^ cells mL^–1^ was aseptically spread on agar plates. The plates were shifted to the temperature-controlled incubator for heat treatment under continuous light of 72 μmol photon m^–2^ s^–1^ PAR provided by fluorescent lamps throughout the heat treatment. The plates were exposed to different temperatures (40–60*^o^*C) for 1 h.

Cells were counted after 3 weeks. LC_50_ was obtained at 150 mM and 45*^o^*C for NaCl and heat, respectively.

### 2.3. Experimental design

**FIGURE 1:**
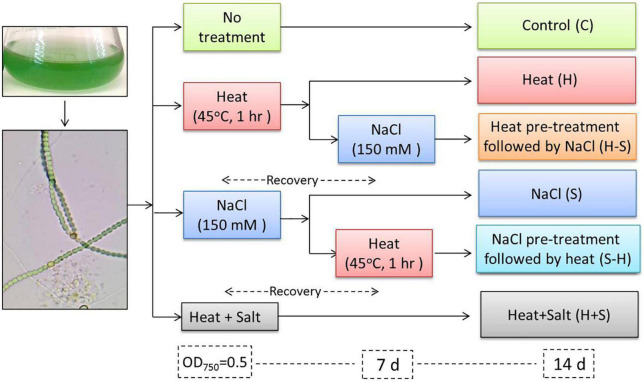
Experimental design.

The experiments were performed in six sets: (1) cells never exposed to stress (C), (2) cells exposed to heat at 45*^o^*C for 1 h (H), (3) cells exposed to salt at 150 mM (S), (4) cells pretreated with heat (45*^o^*C for 1 h) followed by recovery for 7 days and application of salt at 150 mM (H-S), (5) cells pretreated with salt at 150 mM followed by recovery for 7 days and application of heat at 45*^o^*C (S-H), and (6) cells exposed to combined heat and salt stress at respective LC_50_ (H + S) (Figure 1). All the parameters used in the present study were analyzed from the aforementioned cultures. Experiments were performed in triplicates and were repeated two times to confirm the reproducibility.

### 2.4. Growth measurements

Growth was estimated by observing the optical density of the culture at OD_750_ nm in a UV/visible spectrometer (Shimadzu, 2017) on alternate days up to the 15th day using the basal medium as blank (Küpper et al., 2000). It was also measured by cell count through a hemocytometer under a microscope (Olympus, 2019) at 40X on alternate days up to the 15th day (Lawton et al., 1999).

### 2.5. Morphological alterations

The morphological alteration was obtained by taking images of the control culture and culture exposed to stress on 0, 3, 7, and 15 days using a light microscope (Olympus, 2019 model no. CX21iLEDFS1) at 40X, and the image was processed through a computer with Magcam DC-10 camera model and MagVision software (Image calibration: 40X; X:50.000000/40 Micron/Pixel; Y: 50.000000/40 Micron/Pixel).

### 2.6. Pigments estimation

Pigment estimation was done by the protocol of Myers and Kratz (1955). A known volume of cyanobacterial culture was centrifuged and the pellet was incubated overnight at 4*^o^*C in 80% acetone. The suspension was centrifuged and the supernatant was used to measure chlorophyll *a* and carotenoid at 663 and 480 nm, respectively. The pellet thus obtained was used for the estimation of phycocyanin. The pellet was resuspended in 30 mM *Tris–HCl* and sonicated. The suspension was centrifuged and the supernatant was used to measure the phycocyanin content at 610 nm.

### 2.7. Phycobiliproteins spectrum

The supernatant obtained in the pigment estimation for phycocyanin (Myers and Kratz, 1955) was used to study phycobiliproteins spectra by measuring the absorbance from the range of 400–700 nm (Adir and Lerner, 2003).

### 2.8. Malondialdehyde (MDA) estimation

The total content of 2-thiobarbituric acid (TBA) reactive substances expressed as MDA (malondialdehyde) was used to measure lipid peroxidation. These reactive substances were extracted by the protocol of Cakmak and Horst (1991). A known volume of culture was centrifuged and the pellet was suspended in 0.1% of TCA at 4*^o^*C. The suspension was sonicated and centrifuged. A total of 0.5% TBA in 20% TCA was added to the supernatant and the solution was incubated at 90*^o^*C, and then the reaction was terminated in ice-cold water. The suspension was again centrifuged. The absorbance of the supernatant was measured at 532 nm and corrected for non-specific turbidity by subtracting the absorbance at 600 nm. The concentration of MDA was calculated at its extinction coefficient (155 mM^–1^ cm^1^).

### 2.9. Antioxidant assay

Cell pellets of *Anabaena* PCC7120 suspended in lysis buffer (pH 7.0) were sonicated in ice-cold condition. Cell lysis buffer for enzyme assay contained 1 mM EDTA and 1% PVP. The sonicated sample was centrifuged at 4*^o^*C and the resulting supernatant containing crude extract or antioxidant enzymes was used for further assay. Total SOD activity was assayed by monitoring the inhibition of reduction of nitroblue tetrazolium (NBT) according to the method of Giannopolitis and Ries (1977). A 3 ml reaction mixture contained 50 mM potassium phosphate buffer (pH 7.8), 0.1 mM EDTA, 13 mM methionine, 75 μM NBT, enzyme extract, and 2 μM riboflavin. The reaction mixture was illuminated for 20 min at a light intensity of 5,000 μmol photon m^–2^ s^–1^. One unit of SOD activity was defined as the amount of enzyme required to cause 50% inhibition of NBT reduction monitored at 560 nm. Catalase activity was estimated by measuring the consumption of H_2_O_2_ (extinction coefficient 39.4 mM^–1^ cm^–1^) at 240 nm for 3 min (Aebi, 1984). A 3 ml reaction mixture contained 50 mM potassium phosphate buffer (pH 7), 10 mM H_2_O_2_, and enzyme extract. One unit of CAT activity was defined as the amount of enzyme utilized to decompose 1.0 μM of H_2_O_2_.

### 2.10. Transcript analysis

Total RNA extraction using the RNeasy Micro kit (Qiagen) was performed from 50 ml culture (OD_750_nm = 0.6) of *Anabaena* PCC7120 grown in BG11 before and after stress treatment. Total RNA (1 μg) was reverse transcribed in a 20 μl reaction mixture using the iScript cDNA synthesis kit (BioRad). Transcript analysis was performed using gene-specific primer for antioxidative genes, FeSOD, MnSOD, and catalase (*all*0070, *alr*3090, and *alr*2938) as well as reference gene rnpB (Table 1). For qRT-PCR using a CFX-96 (BioRad), 15 ng of cDNA extracted from each sample was used in 20 μl including 10 pmol of each forward and reverse primers, and 1x Sso fast evagreen qPCR supermix (BioRad). Transcript levels were normalized to rnpB transcript and calculated using the 2^–ΔΔ*Ct*^ method to evaluate the relative quantities of each amplified product (Livak and Schmittgen, 2001). The threshold cycle (Ct) was automatically determined for each reaction by the system (default parameters). The specificity of the PCR was determined by melting curve analysis of the amplified products.

**TABLE 1:** Oligonucleotide used for the analysis of the selected antioxidative gene for qRT-PCR.

Gene	Primers
all0070	F: TGGTAGTGGTTGGGTTTGGT R: AGCGTGTTCCCAGACATCAT
alr3090	F: AACGTGGATCAAACAGAGGC R: TCGCTCTCAAATCCCGAACT
alr2938	F: TGATGATGGCGGTACACTGA R: CTGGGCGAGCATTTCTGAAG
rnpB	F: AGGGAGAGAGTAGGCGTTGG R: GGTTTACCGAGCCAGTACCTCT

### 2.11. Statistical analysis

Values presented in the text indicate the mean ± S.E. of the three replicates. The experiments were done in triplicates and repeated two times to confirm the reproducibility. Results were statistically analyzed using two-way ANOVA, followed by Dunnett’s multiple comparisons test to compare changes within groups. A *p*-value of ≤ 0.05 was considered statistically significant. All the statistical analysis was performed using SPSS ver. 22.0 software.

## 3. Results

### 3.1. Growth measurements

**FIGURE 2:**
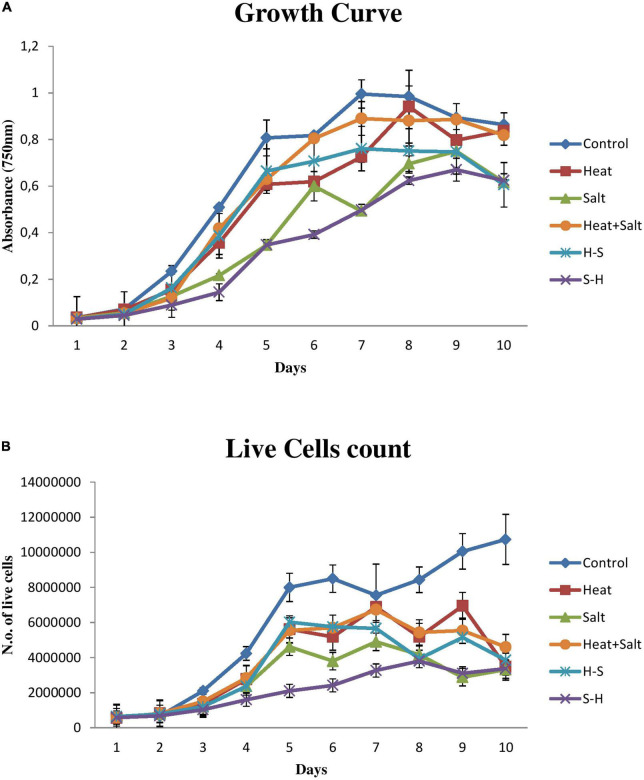
Growth behavior of *Anabaena* PCC 7120 exposed to stress. **(A)** Growth measurement *via* optical density. **(B)** Growth measurement *via* cell count.

The sharp decline in growth can be seen in cells subjected to S-H as shown in Figure 2A, and S-H density declined by 71.57, 56.90, and 50.06% on the 4, 6, and 10th days, respectively. The H-treated sample showed an appreciable decrease from the 6th day to the 10th day of treatment, thereafter there was an increase in its growth. S showed a decrease on the 10th day by 50.47%. However, H+S and pretreated sample, i.e., H-S did not show any stark difference in their growth and were always on par with the control sample. H, H+S, and H-S, did not show any significant change in their cell density. Control bears a sigmoid curve during its growth measurement.

Results pertaining to cell count (Figure 2B) portrayed that the sample treated with S-H was severely affected and showed a sharp decline in its number up to 15 days. H and H+S showed significant cell death by 67.54 and 57.14%, respectively, on the 15th day. S showed significant cell death from the 14th day onward. There was a decline of 71.31 and 69.09% on the 14th and 15 days, respectively. H-S showed a significant decrease on the 12th day by 53.35%, on the 14th day by 48.75%, and on the 15th day by 64.13%.

However, if a correlation has to be drawn between the optical density and cell count, it can be concluded that an increase in cell density does not correspond to an increase in live cell number. An increase in cell density and a decrease in live cell count in a sample can be attributed to the fact that optical density has included the density of live and dead cells together as can be observed in the case of samples treated with H, S, H-S, and H+S.

### 3.2. Morphological alterations

The morphological alterations of *Anabaena* PCC7120 were investigated through light microscopy under various stresses. There were significant differences in the morphological changes shown by the organism under study. Light microscopic studies on the interactions of the stress with *Anabaena* PCC7120 revealed extensive breakage of cyanobacterial filaments.

Significant variation was not observed in samples after 1 h of stress treatment sample and control. Slight yellowing of filaments could be observed in cells subjected to salt stress. Comparative shortening of filaments was observed in all samples on the 3rd day of stress treatment. The akinete formation could be observed in cultures exposed to H + S in order to survive stress conditions. Fragmentation of filaments was observed in the pretreated samples (H-S and S-H).

**FIGURE 3:**
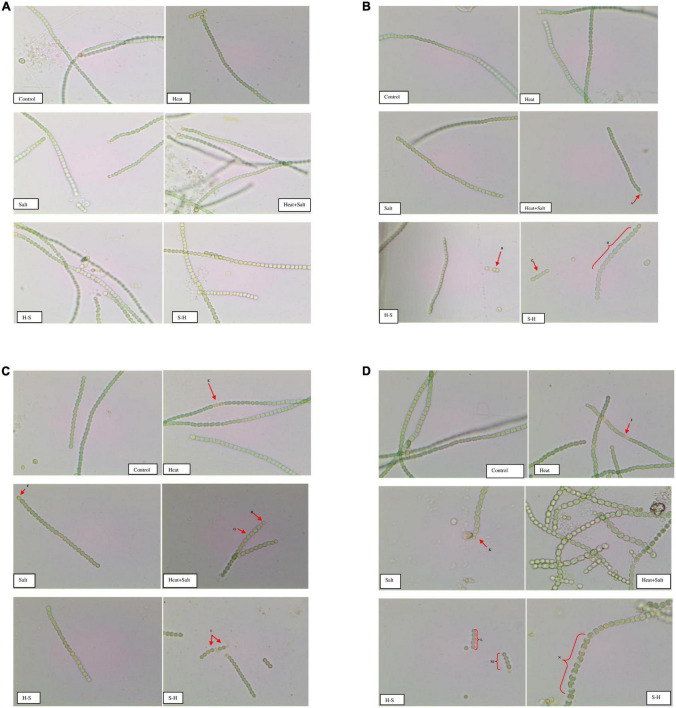
**(A)** Images of *Anabaena* PCC7120 subjected to selected stresses after 1 h (Image calibration: 40X; X:50.000000/40 Micron/Pixel; Y: 50.000000/40 Micron/Pixel). **(B)** Images of *Anabaena* PCC 7120 subjected to selected stresses on 3rd day. (Image calibration: 40X; X:50.000000/40 Micron/Pixel; Y: 50.000000/40 Micron/Pixel. Red arrows or braces indicate a morphological alteration in the filaments. A. Akinete formation; B. and C. Fragmentation of filaments; D. Bulging of cells in filaments). **(C)** Images of *Anabaena* PCC 7,120 subjected to selected stresses on the 7th day (Image calibration: 40X; X:50.000000/40 Micron/Pixel; Y: 50.000000/40 Micron/Pixel. E. & I. Intercalary cell death; F. and H. Akinetes formation; G. Cell bulging). **(D)** Images of *Anabaena* PCC7120 subjected to selected stresses on the 15th day (Image calibration: 40X; X:50.000000/40 Micron/Pixel; Y: 50.000000/40 Micron/Pixel. J. Intercalary cell death; K. Akinete formation; L. and M. Fragmentation of filaments; N. Plasmolysis of cells in filament).

Abnormality in cell shape and size within the filaments was observed in treated cultures on the 7th day. The terminal akinete formation was observed in filaments exposed to stresses. Moreover, prominent intercalary cell death was observed in the culture treated with H and in S-H. Abnormal bulging of cells in the filament of cyanobacterium was observed in H + S. Intercalary cell death appeared in H on the 15th day, whereas cell enlargement and the terminal akinete cell were observed under salt treatment. H + S treatment showed distinctive anomalous cell enlargement in filaments owing to osmotic and heat stress. Fragmentation of filaments was observed in H-S; 4–5 cells/filaments were observed. A peculiar phenomenon of contraction of the protoplast of a plant cell, which may be due to loss of water from the cells, was observed in S-H which resulted in plasmolysis of cells in the filaments due to salinity pretreatment (Figures 3A–D).

### 3.3. Pigment analysis

#### 3.3.1. Chlorophyll *a*

**FIGURE 4:**
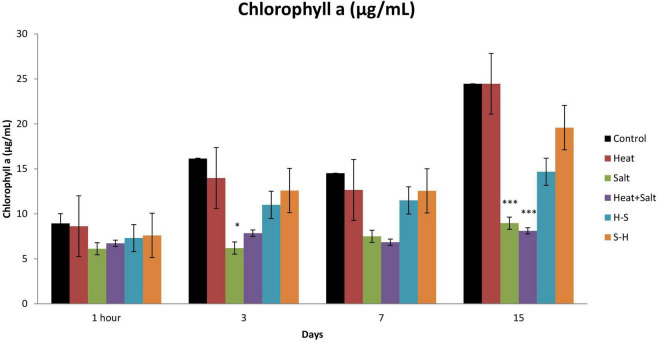
Effect of selected stresses on chlorophyll *a* content of *Anabaena* PCC 7120 up to the 15th day. Asterisk (*) indicates *p* < 0.05 and triple asterisk (^***^) indicates *p* < 0.001. (H-S indicates heat pretreatment; S-H indicates salt pretreatment).

No significant change was observed after 1 h of stress exposure. The chlorophyll content of the culture subjected to treatment declined throughout the observation. On the 3rd day, S showed a significant decline in its chlorophyll concentration by 2.60-fold. Moreover, on the 15th day, cultures subjected to S and H + S treatments showed a significant decrease of 2.73- and 3.02-fold, respectively, in the chlorophyll *a* content. Nonetheless, pretreatment samples showed a decline in the content (approximately 1–1.5-fold change) unlike the other stressed exposed culture but it was on par with the control. It may be that pretreatment had an antagonistic effect on the latter-induced stress (Figure 4).

#### 3.3.2. Carotenoid

**FIGURE 5:**
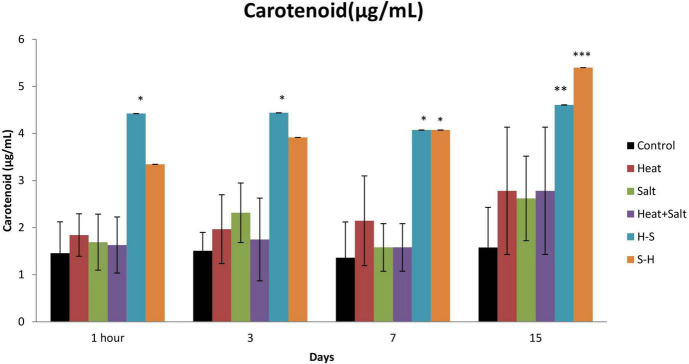
Effect of selected stresses on carotenoid content of *Anabaena* PCC 7120 up to 15th day. Asterisk (*) indicates *p* < 0.05, a double asterisk (^**^) indicates *p* < 0.01, and the triple asterisk (^***^) indicates *p* < 0.001. (H-S indicates heat pretreatment; S-H indicates salt pretreatment).

An increase in carotenoid content was observed throughout all cultures subjected to stress. However, H-S showed a significantly high amount of carotenoid from 1 h up to the 15th day. It showed an increase of 32.94, 33.98, 33.35, and 34.23% on 1 h and 3rd, 7th, and 15th day, respectively, in comparison to the control. Similarly, S-H also showed a significant increase in the amount of carotenoid on the 7 and 15th day (33.35 and 29.19%, respectively) after treatment (Figure 5). The increase of carotenoids in pretreated samples can be attributed as a positive strategy as it protects light-harvesting pigments against photochemical damage.

#### 3.3.3. Phycocyanin

**FIGURE 6:**
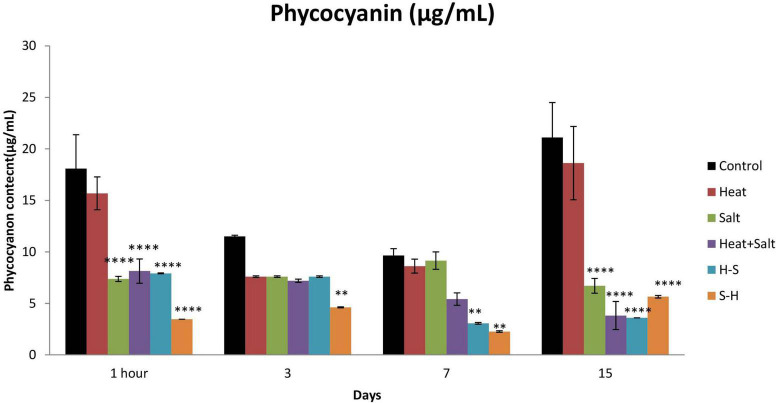
Effect of selected stresses on phycocyanin content of *Anabaena* PCC 7120 up to 15th day. A double asterisk (^**^) indicates *p* < 0.01, the triple asterisk (^***^) indicates *p* < 0.001, and the four asterisk (^****^) indicates *p* < 0.0001. (H-S indicates heat pretreatment; S-H indicates salt pretreatment).

Phycocyanin content was always low for the treated sample up to the 15th day of observation in comparison to the control. There was no significant decline in the phycocyanin content in the H-treated sample. Significant changes were observed in all cultures subjected to stress on 1 h and 15th day except in H, whereas the pretreated sample also showed a significant decrease in the content on the 3rd and 7th day. There was a decrease of 2.45-, 2.22-, 2.29-, and 5.21-fold in S, H + S, H-S, and S-H, respectively, on 1 h after treatment, and 1.13-, 3.15-, 5.52-, and 3.74-fold in S, H + S, H-S, and S-H, respectively, on day 15. S-H showed a decrease in content by 2.49- and 4.426-fold on the 3rd and 7th day, respectively, and H-S showed a decrease of 3.15-fold on day 7 (Figure 6). Pretreatment enhanced the degradation of phycocyanin.

### 3.4. Phycobiliprotein spectrum

**FIGURE 7:**
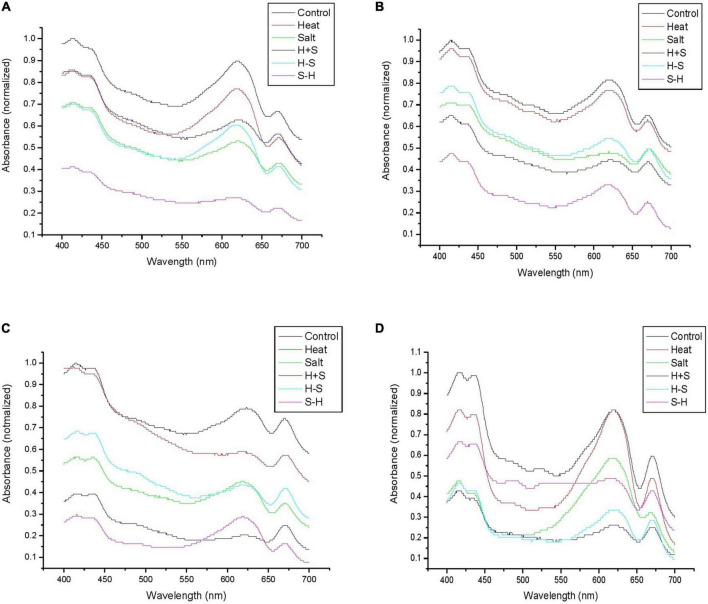
Graph representing the phycobiliprotein spectrum (460–670 nm) of *Anabaena* PCC 7120 exposed to stress on 1 h **(A)**, 3rd **(B)**, 7^th^ **(C)**, and 15th **(D)** day. (H-S indicates heat pretreatment; S-H indicates salt pretreatment).

A study was conducted to assess the effect of stress on phycobiliproteins of *Anabaena* PCC7120. It resulted in a large peak of allophycocyanin (620–660 nm) and phycocyanin (600–640 nm) in control. Phycoeyrthrocyanin peak was also higher in control than in stresses throughout the observation period. The peak area was least affected in H, whereas S-H was severely affected in terms of peak area except on the 15th day. Rest were showing intermediate results. H and H + S showed similar absorption for phycoerythrin and S and H-S also bear close resemblance in peak. A similar result was obtained on the 3rd day with the exception that H + S showed a further decrease in peak. On the 7th day, H was on par with control for the phycoerythrin peak but there was a significant decrease in peak in terms of allophycocyanin and phycocyanin. S and H-S samples were on par with each other in terms of the peak for phycocyanin, whereas the absorption of others remains almost the same as that of the 3rd day. H + S and H-S showed a decrease in their phycocyanin, allophycocyanin, and phycoerythrin peak but S-H showed a large peak on the 15th day (Figure 7). The change in absorption spectra of phycocyanin was similar to the change in the concentration of phycocyanin.

### 3.5. Malondialdehyde (MDA)

**FIGURE 8:**
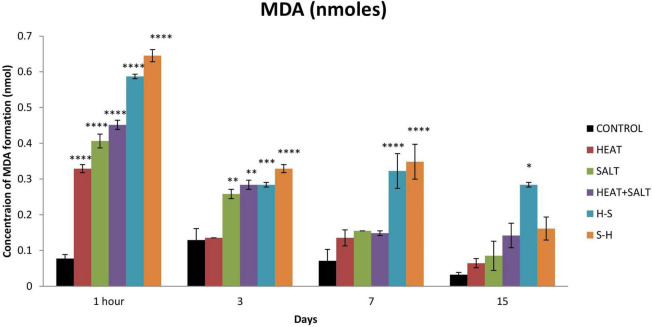
Effect of selected stresses on MDA production in *Anabaena* PCC 7120 up to the 15th day. Asterisk (*) indicates *p* < 0.05, a double asterisk (^**^) indicates *p* < 0.01, the triple asterisk (^***^) indicates *p* < 0.001, and the four asterisk (^****^) indicate *p* < 0.0001. (H-S indicates heat pretreatment; S-H indicates salt pretreatment).

The untreated sample showed a low level of MDA formation throughout the observation, whereas the treated one showed a comparatively high MDA level. MDA formation was significantly increased after 1 h of stress (4.25-fold increase) for H. The MDA content for S gradually increased up to the 7th day (5.25-, 2.66-, and 2.18-fold increase on 1 h and 3rd and 7th day, respectively). H + S showed significant production after 1 h of treatment (5.88-fold increases). H-S and S-H showed a significantly high content of MDA throughout the observation period. In pretreatment, the salt-pretreated sample showed the highest induction of MDA (Figure 8). The high amount of MDA marks high oxidative lipid injury under stress.

### 3.6. Antioxidant assay

#### 3.6.1. Superoxide dismutase

**FIGURE 9:**
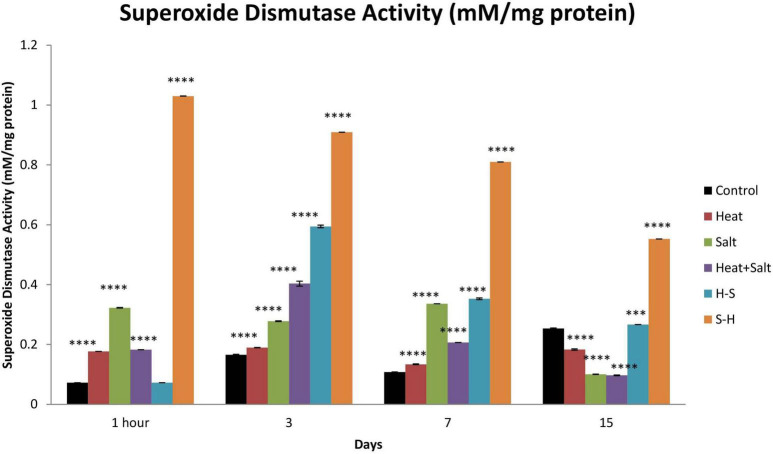
Effect of selected stresses on superoxide dismutase activity in *Anabaena* PCC 7120 up to the 15th day. Four asterisks (^****^) indicate *p* < 0.0001. (H-S indicates heat pretreatment; S-H indicates salt pretreatment).

S-H showed the highest increase in SOD activity throughout the observation with 6.04-, 5.49-, 7.53-, and 2.18-fold increase on 1 h, and 3rd, 7th, and 15th day, respectively, in comparison with control which was followed by H-S. SOD activity was the highest on the 0th day (1-fold), followed by the 3rd day (3.58-fold), the 7th day (3.28-fold), and the 15th day (1.05-fold) for H-S. The least SOD activity among all the treatments shown by H. S showed an increase in activity up to the 7th day (4.45-, 1.67-, and 3.12-fold, respectively). H + S displayed an increase in its SOD activity on 1 h and 3rd day by 2.52- and 2.43-fold, respectively (Figure 9).

#### 3.6.2. Catalase

**FIGURE 10:**
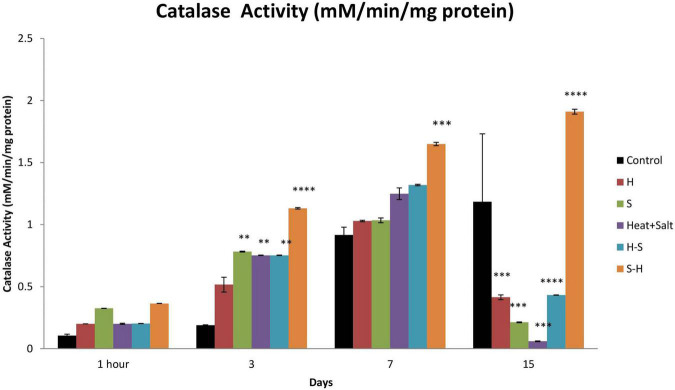
Effect of selected stresses on catalase activity in *Anabaena* PCC 7,120 up to the 15th day. A double asterisk (^**^) indicates *p* < 0.01, the triple asterisk (^***^) indicates *p* < 0.001, and the four asterisk (^****^) indicates *p* < 0.0001. (H-S indicates heat pretreatment; S-H indicates salt pretreatment).

No significant changes were observed in catalase activity after 1 h of treatment. Catalase activity showed maximum induction throughout the observation in S-H. There were 5.96-, 1.79-, and 1.61-fold increase on 3rd, 7th, and 15th day, respectively. H-S showed an increase in its activity up to the 7th day with a significant increase on the 3rd day (3.97-fold). S and H + S showed similar activity to that of heat pretreatment. However, no significant increase in catalase activity was shown by H (Figure 10).

### 3.7. Transcript analysis

**FIGURE 11:**
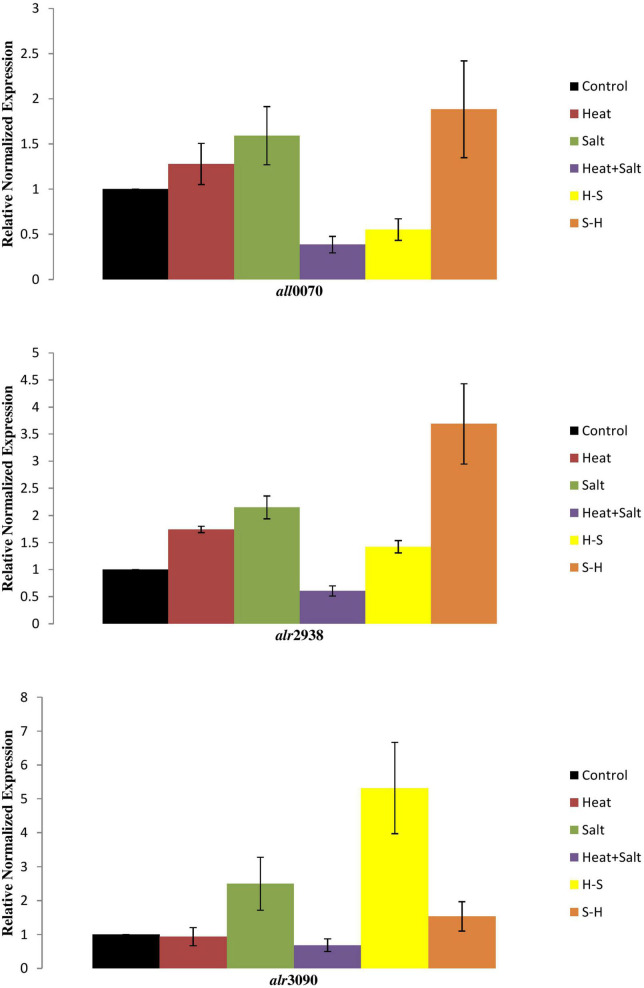
Transcript level of cyanobacterial genes under stresses.

Expression of three genes of the antioxidative defense system (FeSOD, MnSOD, and catalase) was observed in response to selected stresses, which displayed differential accumulation as compared to control. The expression of the genes *alr*2938 and *all*0070, which, respectively, code for FeSOD and MnSOD, increased by 3.6- and 1.8-fold in salt-pretreated (S-H) samples, indicating activation of stress defense pathways in response to ROS generation, supporting our biochemical findings. Likewise, cells exposed to H and S also displayed accumulation of *alr*2938 and *all*0070. Transcript levels of *all*0070 were downregulated under H-S and H + S samples. Similarly, catalase (*alr*3090) expression was also downregulated in H and H + S samples, whereas increased in S, S-H, and H-S samples (Figure 11).

## 4. Discussion

The present study deals with the effect of heat shock, salinity, their combination, and pretreatment stresses on growth, morphological alteration, pigments, lipid peroxidation, and antioxidant enzymes of *Anabaena* PCC 7120.

Morphological alterations through light microscopy demonstrated extensive shrinkage of cyanobacterial filament, cell degeneration, and terminal akinetes formation. It further revealed that the heat initially had no impact on the cellular structure, however, significant cell degeneration was observed in later phases. A similar result was observed under salinity stress, but anomalous swelling of cells was a unique feature observed on the 15th day, which could be due to osmotic stress leading to the accumulation of osmoprotectant (Singh and Montgomery, 2013). Heat pretreatment showed fragmentation of the filaments. H + S showed abnormal bulging of the cells in the filaments, which could be attributed to the fact that in addition to the cellular toxicity brought on by high ion concentrations, high salt concentrations in the growth media are known to reduce the water potential (Singh and Montgomery, 2013). Osmotic stress is thus caused by salt stress, and under these circumstances, many organisms, including cyanobacteria, store or synthesis suitable solutes or osmoprotectants to maintain reduced water potential inside the cell (Singh and Montgomery, 2013). In S-H, plasmolysis of cells in the filaments due to salinity pretreatment followed by heat was observed, which may be due to the hypertonic environment of the cyanobacterium. Similar to past observations on the response of cyanobacteria to abiotic stress conditions, particularly nitrogen constraint, akinete formations were observed. These findings require in-depth analysis because they are intriguing (Mahawar et al., 2018).

The decline in the chlorophyll *a* content in stressed samples could be attributed to the production of ROS that caused bleaching due to active oxygen-mediated peroxidation (Rai et al., 2021). Moreover, the comparative increase in the content of pretreated samples reflects the occurrence of cross-tolerance of heat-pretreated and salt-pretreated *Anabaena* to salt and heat stress, respectively. This result was concordant with the study of Mishra et al. (2009), which reported heat-mediated alleviation of UV-B toxicity in *Anabaena doliolum.*

The result of the pretreatment effect on carotenoid content can be further supported by the study of Mishra et al. (2009), where they reported an increase in carotenoid content on heat shock pretreatment followed by UV-B treatment. An increase in carotenoids in *Anabaena* PCC7120 under stress can be attributed to a positive strategy (Nonnengiesser et al., 1996). It is due to its role of protecting light harvesting pigments in the antenna complex against photochemical damage by allowing triplet energy transfer from chlorophyll *a* to carotenoid (Kim et al., 2005). They exert photoprotective and antioxidative functions by dissipating excess energy as heat by non-photochemical quenching (NPQ) or by scavenging excess ROS (El Bissati et al., 2000). Moreover, carotenoids are potent non-enzymatic antioxidants, which scavenge a variety of reactive oxygen species when cyanobacteria are exposed to stress. They play a role of a modulator of membrane homeostasis and photosynthetic apparatus against abiotic stress (Kirilovsky and Kerfeld, 2016).

Phycocyanin content significantly decreased under all treatments except heat up to the 15th day of observation compared to the control. The decrease in phycocyanin under S, H + S, H-S, and S-H may be due to the rapid entry of sodium ions leading to the dismantling of phycobilisomes from the thylakoid membranes causing photosynthesis inhibition and reduced energy transfers from phycobiliproteins to the reaction center (Lu and Vonshak, 2002; Rafiqul et al., 2003). Similar results have been earlier reported by Hemlata and Fatima (2009) on *Anabaena* NCCU-9, Singh et al. (2012) on *Anabaena* sp. PCC 7120, and Verma and Mohanty (2000) on *Spirulina platensis*, thus supporting present findings. Samples exposed to salt pretreatment were the most affected followed by heat pretreatment. Moreover, an increase in the intracellular concentration of NaCl inactivates ATP synthase and decreases the intracellular level of ATP (Murata et al., 2007). Moreover, ROS-induced protein peroxidation may also cause a damaging effect on phycocyanin.

Phycobiliproteins are water-soluble proteins that are covalently bonded with phycobilins (Hu, 2019). Due to phycobilins, phycobiliprotein have distinct absorption spectra from 460 to 670 nm (Adir and Lerner, 2003). Phycobiliproteins are divided into three different classes based on phycobilin energy or absorption spectra. Phycoerythrin (PEs) or phycoerythrocyanin (PECs) are of higher energy with main absorption at 480–580 nm, phycocyanin (PCs) of intermediate level with absorption at 600–640 nm, and allophycocyanins (APCs) with absorption at 620–660 nm. The change in the spectrum can be attributed to the fact that stresses led to photosynthetic inhibition. Inside the photosynthetic apparatus, the phycobilisome, which is involved in light capture, may be a target of photodamage and oxidative toxicity. These results find support from the studies of Wen et al. (2005) and Zhang et al. (2010) on *Spirulina platensis* under salt and heat stress, respectively.

Lipids play an important role in the tolerance to several physiological stressors in a variety of organisms including cyanobacteria (Parida and Das, 2005). An increase in the level of lipid peroxidation under various stresses can be attributed to the increased production of reactive oxygen species indicating oxidative stress in cyanobacteria, which can lead to cellular damage (Rai et al., 2021). In cyanobacteria, the membrane contains a high amount of polyunsaturated fatty acid (PUFA) (Halliwell, 1999), and increased MDA content is due to oxidative degradation of PUFA (Girotti, 1990). Increased lipid peroxidation in salt pretreatment (S-H) could be due to salinity-induced membrane damage, which was followed by heat treatment and enhanced lipid peroxidation. This finds support from the work of Srivastava et al. (2006), where a similar increase was reported in salt-pretreated samples followed by UV-B in *Anabaena doliolum*.

In order to scavenge stress-induced ROS, cyanobacteria activate their antioxidative defense system. SOD activity followed a general trend of S-H > H-S > S > H > C except on the 15th day of treatment suggesting recovery and suppression of the antioxidative defense system on the 15th day. Cyanobacteria induce a complex antioxidant defense system to combat the increasing level of ROS. SODs are the first line of defense against ROS. Superoxide dismutase is induced by a variety of cyanobacteria under stress and dismutates the superoxide radical (Zutshi et al., 2008). Superoxide dismutase is a major oxygen-free radical scavenger and it functions by disproportionating free radicals into hydrogen peroxide which is further scavenged by catalase (Macháèková, 1995).

A similar result was obtained for catalase, where pretreatment enhanced the activity. Peroxide radical produced by SOD during the scavenging of free radicals is further scavenged by catalase (Halliwell and Gutteridge, 2015). Catalases belong to the category of well-characterized antioxidant enzymes for hydroperoxide detoxification that cleaves the peroxide bond in hydrogen peroxide (H–O–O–H) to form molecular oxygen and water (Srivastava, 2010). Hence, it appears that peroxide can be detoxified by CAT.

Biochemical findings were further strengthened after analyzing the transcript levels of respective genes. Our findings imply that salinity pretreatment has an impact on the transcriptional control of genes involved in antioxidant production. Surprisingly, at heat pretreatment, the transcript levels of FeSOD and MnSOD were significantly downregulated. This may imply that the production of FeSOD and MnSOD is not alarm-driven by ROS or other cellular signals of temperature stress. Nevertheless, catalase gene expression was elevated. However, enhanced FeSOD and MnSOD under salt pretreatment (S-H), as compared with salinity, indicate an adaptive strategy of *Anabaena* for scavenging excessive ROS produced by the cyanobacteria.

## 5. Conclusion

**FIGURE 12:**
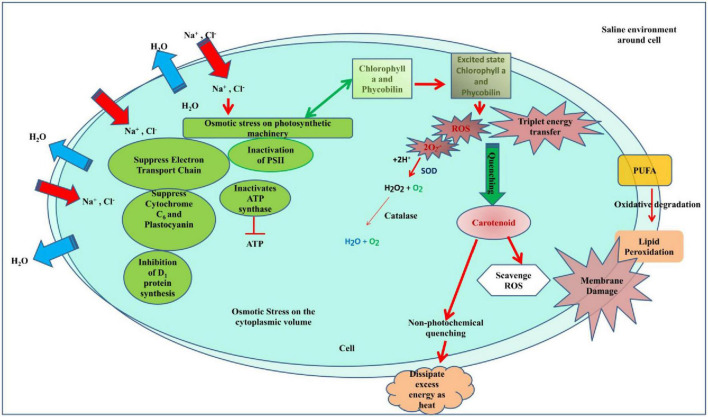
Hypothetical model showing how salinity pretreatment impairs the cell equilibrium and metabolism which when exposed to heat shock makes the cell more fragile.

The study was conducted to analyze the effect of stresses on cyanobacteria with the main aim to examine the effect of pretreatments on growth, development, pigment, lipid peroxidation, and antioxidant activity. Cyanobacteria were chosen due to their cosmopolitan nature, and particularly, *Anabaena* PCC7120 was chosen as a model organism as it bears a close resemblance to higher plant and easy maintenance of the culture in the laboratory. The cyanobacterium was able to effectively detoxify the surplus reactive oxygen species formed inside the cells of stressed *Anabaena* PCC7120 due to the enhanced expression of SOD in salt pretreatment. It can be concluded that the salinity pretreatments enhanced the deleterious effect. Salt was found to synergize the effect of heat on cyanobacteria as it caused osmotic stress in the cells that affect the cellular equilibrium whereas heat has been found to offer some cross tolerance to abiotic stresses due to the production of heat shock proteins. Heat shock proteins may be responsible for the protective effect of heat against salt. Pretreatment at sublethal temperatures increases the thermotolerance of cyanobacterial species, both unicellular and filamentous, and suggests that heat shock genes and proteins are involved in thermotolerance. Due to the constant synthesis of the two Hsp60 proteins throughout the heat stress and their great stability even after returning to normal growth circumstances, *Anabaena* demonstrates exceptional recovery from heat stress. On the other hand, salinity increased heat toxicity. The cellular equilibrium of the cell is disturbed by salt. Pretreatment with salinity impairs membrane permeability. In addition, it interferes with the cell’s ideal environment, which hinders enzyme activity, and is further hampered by heat shock (Figure 12). When exposed to heat, such pretreated cells become more fragile. Hence, it can be inferred that when physical and chemical stresses are superimposed 7 days apart, pretreatment chemical stress synergizes the deleterious effect of physical stress but pretreatment physical stress offers some tolerance to chemical stress. Therefore, this study is the first to provide how cyanobacteria respond in the presence of physical and chemical stresses and that chemical stress enhances the damaging effect of physical stress.

## Data availability statement

The original contributions presented in this study are included in the article/supplementary material, further inquiries can be directed to the corresponding author.

## Author contributions

RS and NA conceived the idea and designed the experiments. RS conducted the experiments. VK performed the transcript analysis. RS, TK, SaY, NS, ShY, RP, VK, and NA analyzed the data. RS and ShY wrote the manuscript with input from other co-authors. All authors contributed to the article and approved the submitted version.
